# Music modulates emotional responses in growing pigs

**DOI:** 10.1038/s41598-022-07300-6

**Published:** 2022-03-01

**Authors:** Juliana Zapata Cardona, Maria Camila Ceballos, Ariel Marcel Tarazona Morales, Edimer David Jaramillo, Berardo de Jesús Rodríguez

**Affiliations:** 1 Grupo de Investigación QUIRON, Escuela de Medicina Veterinaria, Universidad de Antioquia, Calle 70 No. 52-21, Medellín, Colombia; 2 Department of Production Animal Health, Faculty of Veterinary Medicine, University of Calgary, Clinical Skills Building, 11877–85th Street NW, Calgary, AB T3R 1J3 Canada; 3 Grupo de Investigación BIOGEM, Departamento de Producción Animal, Facultad de Ciencias Agrarias, Universidad Nacional de Colombia, Cra. 65 No. 59A - 110, Medellín, Colombia; 4 Grupo de Investigación Nutri-Solla, SOLLA S.A., Carrera 42 # 33 – 80, Itagüí, Colombia

**Keywords:** Emotion, Animal behaviour

## Abstract

There is a lack of clarity on whether pigs can emotionally respond to musical stimulation and whether that response is related to music structure. Qualitative Behavioral Assessment (QBA) was used to evaluate effects of 16 distinct musical pieces (in terms of harmonic structure) on emotional responses in nursery pigs (n = 30) during four periods: “habituation”, “treatments”, “breaks” and “final”. Data were evaluated using Principal component analysis (PCA). Two principal components (PC) were considered in the analysis: PC1, characterized as a positive emotions index, included the emotional responses content, playful, sociable, and happy, whereas PC2, characterized as a negative emotions index, included fearful, inquisitive, and uneasy with positive loadings, and relaxed and calm with negative loadings. Musical stimulation (treatment) increased (*P* < 0.01) both emotional indices, compared to other periods and this response was influenced by harmonic characteristics of the music. We concluded that pigs have a wide variety of emotional responses, with different affective states related to the music structure used, providing evidence of its potential use as environmental enrichment for this species.

## Introduction

The use of music and its effects on emotions has been broadly studied in humans, with indications that the type of acoustic stimulation influences both mood^1,2^ and behavior^3,4^, inducing a wide variety of affective experiences in humans^5^. Thus, music has been recognized as a powerful emotional communication tool^6^. However, with scientific recognition of sentience in animals^7^ the need for research in non-human models has emerged, to understand emotions from biological and evolutionary perspectives^8,9^.

Auditory signals in animals are useful to communicate with recipients^10,11^ or to manipulate their behavior^11,12^. Studies on the effect of music on biological responses have been performed, reporting increased milk production in cows^13^ and growth rates in carp^14^. Other studies focusing on behavioural responses have reported various effects, including decreased abnormal behaviors including fewer stereotypies in elephants^15^ and reduction of aggression and agitation in chimpanzees^16^; decreased anxious behavior in gorillas^17^; and reduction of barking and increased periods of resting in dogs^18^. Furthermore, depending on the music presented, changes in activity have been reported in pigs^19^ and tamarins^20^. However, to our knowledge, there are no studies on non-human animals focusing on evaluation of emotional responses to music.

Previous studies demonstrated that specific acoustic features are effective to induce behavioral or physiological effects in non-human animals^21,22^. An important principle is that music constitutes a signal comprised of multiple space–time acoustic elements and non-human animals can differentiate basic structural components of music, e.g., tempo, rhythm, and tonality^23–25^, with important differences between species in their perception of various acoustic parameters^26^. In humans, consistent emotional responses to music can be achieved through harmony, a structural property considered as "an emotional characteristic"^27,28^. Neuropsychological studies supported this hypothesis, associating this musical characteristic with specific brain activity in areas related to processing various emotions^29^. Harmony is defined as consonant or dissonant. Consonant patterns lead to calmness and relaxation feelings, whereas dissonance induces anger or fear feelings ^22^. It has been hypothesized that the understanding of those patterns is also present in non-human animals^30,31^. Although research in this field in non-human animals is still scarce, a predilection for consonant music has been suggested in some primates and chickens^32,33^. Therefore, the importance of the type of music^34^ and aspects like the harmonic structure have been raised as a determinant in the potential effects of music in animals^22^.

There is a knowledge gap about the effects of specific music characteristics, e.g., harmony, on animals’ emotions. Therefore, in this study, we exposed nursery pigs to various types of music (in terms of harmony), and compared emotional states with periods without music using QBA. Thus, we evaluated effects of musical stimulation and the influences of its structure on the modulation of emotional responses. Our results are expected to provide a basis for the design and application of music as environmental enrichment for animals.

## Results

### Emotional responses to musical stimulation

The states frustrated, apathetic, distressed, and bored were not observed during any period of the stimulation protocol and therefore not included in analyses. The state fearful was exclusively observed during treatment periods. States agitated and inquisitive were reported only during treatment and breaks, with no presentation during other periods. Uneasy was not observed during the habituation period. The distribution of emotional responses at various periods are presented (Table 1).

**Table 1:** Descriptive data of pigs’ scores (in cm) to each QBA emotional state [mean, standard deviations (SD), maximum (max), minimum (min) and coefficient of variation (CV)] during each period of the musical stimulation protocol.

Emotional response	Moments															
	Habituation				Treatment				Break				Final			
	Mean ± SD	Min	Max	CV	Mean ± SD	Min	Max	CV	Mean ± SD	Min	Max	CV	Mean ± SD	Min	Max	CV
Active	4.1 ± 1.7	2.5	7	41.7	6.9 ± 3.5	0.5	12.5	50.2	3.9 ± 1.7	1.0	7.0	41.6	2.0 ± 1.5	0.5	4	76.5
Relaxed	3.3 ± 2.3	0.0	5.6	69.3	2.6 ± 2.7	0.0	11.0	101.9	3.4 ± 1.7	0.4	5.3	49.3	4.6 ± 2.1	1.5	6.7	44.6
Fearful	0.0	0.0	0.0	0.0	0.8 ± 1.5	0.0	6.4	192.2	0.00	0.0	0.0	0.0	0.0	0.0	0.0	0.0
Agitated	0.0	0.0	0.0	0.0	2.3 ± 3.6	0.0	12.1	154.3	0.1 ± 0.3	0.0	1.5	425	0.0	0.0	0.0	0.0
Calm	3.6 ± 0.9	2.3	4.8	27.1	2.4 ± 2.9	0.0	12.0	117.2	3.4 ± 1.3	1.0	5.0	37.5	4.3 ± 1.2	2.8	5.8	28.7
Content	1.2 ± 0.6	0.6	2	49.2	3.5 ± 3.5	0.0	9.5	98.3	1.4 ± 0.8	0.4	3.4	54.3	0.7 ± 0.5	0.0	1.4	79.4
Indifferent	4.0 ± 0.7	3	5	17.8	0.9 ± 2.0	0.0	8.0	232.6	1.1 ± 0.9	0.0	2	77.3	1.8 ± 2.0	1.0	2	25
Friendly	1.3 ± 1.2	0.4	2.9	90.2	3.2 ± 3.3	0.0	10.5	105.1	1.5 ± 0.9	0.0	3.4	64.7	0.9 ± 1.0	0.0	2.6	111.1
Playful	1.3 ± 0.9	0.4	2.5	67.2	3.5 ± 3.5	0.0	11.5	100	1.1 ± 0.8	0.0	2.7	74.3	0.7 ± 0.8	0.0	2	123.5
Positively occupied	2.6 ± 0.9	1.2	3.4	36.9	3.4 ± 2.6	0.0	11.5	74.8	3.9 ± 1.3	1.5	6.0	32.7	2.3 ± 1.9	0.7	4.5	85.1
Lively	2.8 ± 0.5	2	3.2	16.9	5.4 ± 2.73	0.0	5.45	50.1	3.5 ± 1.1	1.2	5.0	32.7	1.5 ± 1.9	0.0	4.5	128.6
Inquisitive	0.0	0.0	0.0	0.0	2.6 ± 3.4	0.0	10.5	132.6	0.4 ± 0.8	0.0	2.5	190.2	0.0	0.0	0.0	0.0
Irritable	0.3 ± 0.6	0.0	1.3	223.1	0.6 ± 1.1	0.0	4.8	183.6	0.2 ± 0.4	0.0	1.4	262.5	0.0	0.0	0.0	0.0
Uneasy	0.0	2.3	4.8	27.1	0.7 ± 1.7	0.0	12	117.2	0.1 ± 0.4	1.0	5.0	37.5	0.2 ± 0.4	2.8	5.8	28.7
Sociable	1.5 ± 0.7	0.7	2.5	46.7	3.2 ± 3.2	0.0	11	98.8	1.5 ± 0.8	0.0	3.0	55.9	1.0 ± 0.9	0.0	2.5	96
Happy	0.8 ± 0.2	0.6	1.0	20.0	3.3 ± 3.2	0.0	12	97.6	0.9 ± 0.7	0.0	2.4	66.3	0.6 ± 0.4	0.0	0.8	58.3

States active, fearful, agitated, calm, content, friendly, playful, lively, inquisitive, sociable, and happy had higher averages during the treatment period compared to habituation, breaks, and final periods. States calm and relaxed had the highest averages in the final period. Indifferent had a higher average during habituation and positively occupied during breaks. In general, standard deviations reported for various states were higher during treatments, indicating a wide range in the rating of emotional responses during exposure to music.

PCA generated two principal components (PC) with eigenvalues exceeding 1.5. These PC explained a total of 66.12% of the variance among variables. In the first principal component (covering 44.45% of the variance), states active, agitated, content, friendly, playful, positively occupied, lively, sociable, and happy had positive loadings above 0.6, and this factor was characterized as a positive emotions index. In the second component (explaining 21.67% of the variance), two states (relaxed and calm) had negative loadings above 0.6, and three states (fearful, inquisitive and uneasy) had positive loadings above 0.6, and this factor was characterized as a negative emotions index, as the higher this index, the more negative emotions the pigs had (Table 2).

**Table 2:** Principal component analysis.

Emotional response	PC1 Positive emotions index	PC2 Negative emotions index
Active	**0.84**	0.38
Relaxed	0.09	**− 0.79**
Fearful	− 0.15	**0.71**
Agitated	**0.62**	0.48
Calm	0.11	**− 0.78**
Content	**0.94**	0.02
Indifferent	− 0.42	− 0.41
Friendly	**0.90**	− 0.15
Playful	**0.95**	0.02
Positively occupied	**0.73**	− 0.19
Lively	**0.82**	0.04
Inquisitive	− 0.13	**0.79**
Irritable	0.43	0.35
Uneasy	− 0.26	**0.60**
Sociable	**0.91**	− 0.14
Happy	**0.95**	− 0.07
% of variance	44.45	21.67

Loadings for the 16 emotional states of qualitative behavior assessment (QBA). Loadings greater than 0.6 are bolded and were used to define the indexes identified in the analysis.

**Figure 1:**
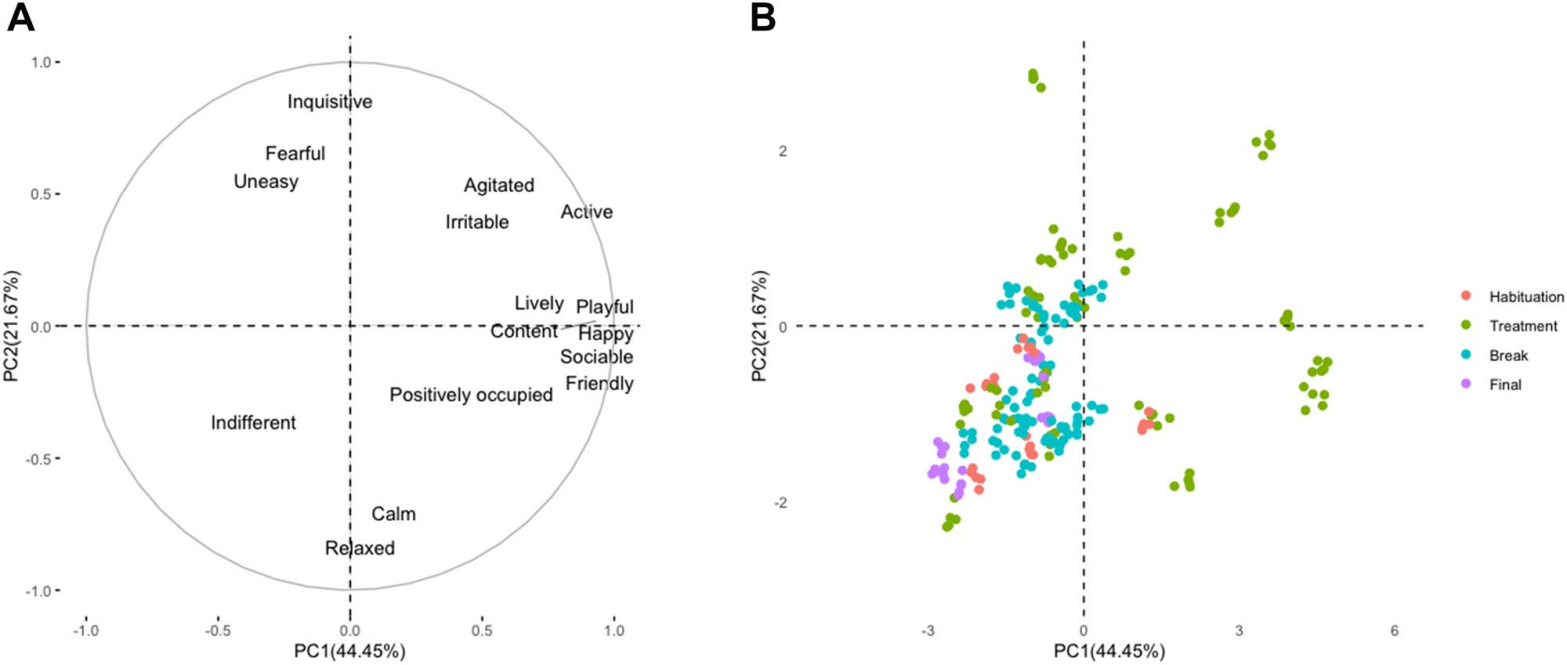
(**A**) Plots of loadings for qualitative behavior assessment (QBA). Emotional responses in dimensions PC1 (positive emotions) and PC2 (negative emotions). (**B**) Individual loadings associated with the four evaluated periods. Colors refer to responses on each period: habituation (red), treatment (green), break between musical pieces (blue), and final (purple).

During treatment, both positive and negative emotions were expressed and widely distributed in the four PCA quadrants. In contrast, responses for breaks, habituation, and final periods were densely grouped, with little variability, occupying mostly a single quadrant (IV) and occasionally quadrant I (see Fig. 1B). Furthermore, the location of these observations indicated that during these periods, the emotional responses were predominantly calm, relaxed, and indifferent (see Fig. 1A). Figure 1 summarizes PCA results and displays the relationship between the states contributing to each component and individual responses for all evaluated periods.

Positive and negative emotion indexes differed (*P* < 0.05) between evaluated periods. Treatment differed from habituation (*P* = 0.005), breaks (*P* = 0.005) and final (*P* = 0.0003) periods in the positive emotions index, with higher values for treatment. There were no differences (*P* > 0.05) among other periods. Similarly, in the negative emotions index, treatment period differed from others (*P* values = 0.00009, 0.001, and 0.0002, respectively), with higher values, but there were no differences among other periods (*P* > 0.05) (Table 3).

**Table 3:** Adjusted means (± SEM) and confidence interval (ICC) of the positive and negative emotion PCA indexes on habituation, treatment, break and final periods of the musical stimulation protocol.

Index	Periods								Difference between periods *P* value
	Habituation		Treatment		Break		Final		
	Mean ± SEM	ICC (Lower limit–Upper limit)	Mean ± SEM	ICC (Lower limit–Upper limit)	Mean ± SEM	ICC (Lower limit–Upper limit)	Mean ± SEM	ICC (Lower limit–Upper limit)	
Positive emotions	− 1.60^b^ ± 0.45	[− 1.98, − 0.13]	1.22^a^ ± 0.31	[0.58, 1.86]	− 0.97^b^ ± 0.23	[− 1.44, − 0.49]	− 1.99^b^ ± 0.45	[− 2.92, − 1.07]	< 0.001
Negative emotions	− 1.01^b^ ± 0.33	[− 1.66, 0.36]	0.91^a^ ± 0.14	[0.63, 1.19]	− 0.66^b^ ± 0.17	[− 0.98, − 0.33]	− 1.27^b^ ± 0.33	[− 1.92, − 0.62]	< 0.001

^ab^Within a row, means without a common superscript differed (*P* < 0.05).

SEM, standard error of the means; ICC, confidence interval.

### Music type effect

**Figure 2:**
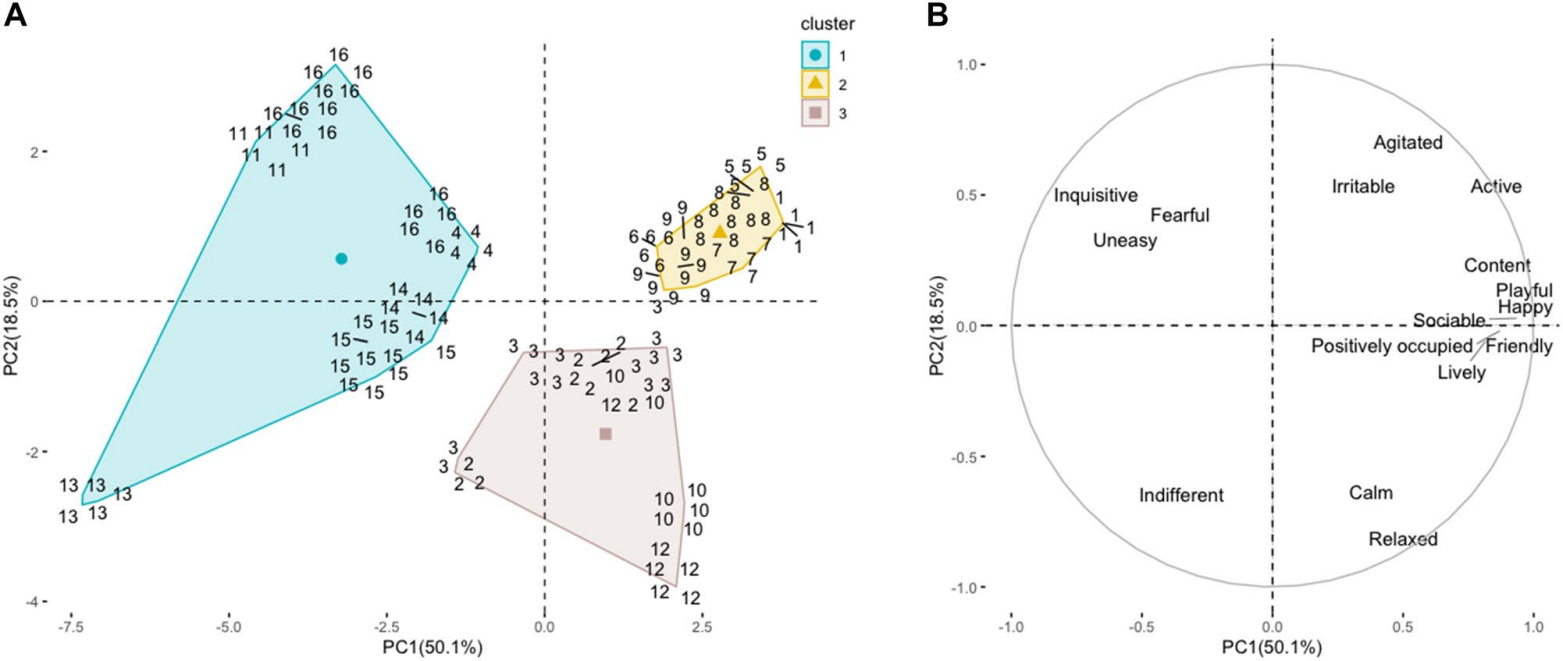
Cluster analysis. (**A**) Similarity between musical pieces according to cluster analysis of the k-means method (K = 3); each cluster included pieces homogeneous within themselves and heterogeneous among themselves. We recognized three clusters: Group 1 (blue), which includes dissonant pieces; Group 2 (yellow) corresponds to consonant pieces; and Group 3 (brown) corresponds to pieces without harmony. (**B**) Plots of loadings for qualitative behavior assessment (QBA) emotional states in dimensions PC1 and PC2.

A cluster analysis was conducted to evaluate the relationship between musical pieces and animals' emotional responses (Fig. 2). Three clusters were selected with the k-means method. Pieces that conformed to each cluster coincided with the different musical groups of compositional harmonic characteristics. Cluster 1 (pieces 4, 11, 13, 14, 15 and 16), coincided with dissonant harmonic structure (Group 1); Cluster 2 (pieces 1, 5, 6, 7, 8 and 9) corresponded to consonant harmony (Group 2); and Cluster 3 (pieces 2, 3, 10, and 12) corresponded to pieces without harmony (Group 3).

Positive and negative emotion indexes differed (*P* < 0.0001) between evaluated clusters (Table 4). Consonants pieces were grouped in Cluster 2 (quadrant II, see Fig. 2A,B), and had higher values for the positive emotions index compared to cluster 1 (*P* < 0.0001) and Cluster 3 (*P* < 0.0001), that did not differ (*P* = 0.07). In contrast, dissonant pieces grouped in Cluster 1, had the highest values for the negative emotion index compared to Cluster 2 (*P* = 0.0059) and Cluster 3 (*P* = 0.0002), that did not differ (*P* = 0.13). Consistently, when contrasting Cluster 1 with the PCA plot, they were located mainly in quadrant I with observed states such as fearful and uneasy (see Fig. 2A,B). Pieces without harmony (Group 3) were located in quadrants III and IV and related with emotional responses calm, relaxed, and indifferent.

**Table 4:** Generalized linear mixed models of the components in clusters formed by musical pieces.

Index	Cluster						Difference between clusters *P* value
	1		2		3		
	Mean ± SEM	ICC (Lower limit–Upper limit)	Mean ± SEM	ICC (Lower limit–Upper limit)	Mean ± SEM	ICC (Lower limit–Upper limit)	
Positive emotions	− 2.74^b^ ± 0.31	[− 3.37, − 2.11]	1.74^a^ ± 0.26	[1.20, 2.28]	− 1.10^b^ ± 0.43	[− 1.99, − 0.21]	< 0.001
Negative emotions	0.65^a^ ± 0.26	[0.11, 1.19]	− 0.10^b^ ± 0.19	[− 0.50, 0.29]	− 0.92^b^ ± 0.37	[− 1.68, − 0.15]	< 0.001

^ab^Within a row, means without a common superscript differed (*P* < 0.05).

SEM, standard error of the means.

## Discussion

Pigs are able to discriminate familiar people, strangers, and objects based on visual, auditory, and olfactory signals^35,36^. In addition, pigs have auditory sensitivity similar to primates, being able to distinguish between tones of different frequencies^37^. These cognitive and sensory characteristics make the species particularly interesting as a model for studying effects of music on emotions. Furthermore, results obtained herein provided additional evidence on the ability of pigs to emotionally respond to musical stimulation and the potential of various musical characteristics to induce different emotional responses in animals.

### Emotional responses to musical stimulation

Emotionality seems to be the natural way to evaluate effects of music, at least in humans; although music affects cortical cognitive pathways, it also affects subcortical regions of our brains, related to induction of emotions^38^. Furthermore, various brain systems mediate emotions such as anger, fear, joy, sadness^39^, and a variety of social emotions^40^, and there is evidence that music can activate the body within the framework of a specific emotion^41^. Several studies reported heterogeneous results in the evaluation of effects of music on behavioral responses and productive indicators in various species, including nonhuman primates, dogs, cows, chickens, carps, elephants, tamarins and pigs^13–20,42,43^. However, there is a lack of studies assessing emotional responses. Therefore, the evaluation carried out herein focused on affective responses. We evaluated four experimental periods (with and without musical stimulation) and their effects on emotional responses in pigs, demonstrating remarkable differences.

During the treatment period, the mean for each QBA emotional response (except for relaxed, calm, indifferent, and uneasy) was higher compared to periods without music, and also had a greater standard deviation; therefore, we inferred that expression of emotional responses in the pigs was more intense and diverse during exposure to music. Even negative states like fearful were exclusive to treatment periods, absent even in breaks, which immediately followed a musical piece, so its presentation was directly attributed to musical stimulation.

Based on PCA analysis, according to emotional valences there was a clear separation of QBA states into two groups: positive and negative emotional indexes. There were significant differences during the music exposition in states of positive and negative emotion indexes compared to other periods. Based on our results, we inferred that pigs responded to a piece of music. There is evidence and theoretical perspectives indicating that various brain areas may be critical for processing and appreciate music emotionally, and we (humans) share them in a homologous way with all other mammals^6,38,40^. Our findings are novel because effects of musical exposure on the emotion of domestic pigs have not been investigated, and the few existent reports on the species have focused on behavior, with inconsistent results. Studies have reported music-induced behavioral changes in resting or exploration^19,44^; however, another study did not report any effect^45^.

There was a higher level of relaxation in the pigs once musical stimulation was finished; this was explained by increased activity during treatments, where they were busy performing social behaviors, including play. Similar results during exposure to music have been reported. Pigs exposed to musical stimulation after weaning affected “resting;” therefore, music influenced playing behavior at the expense of resting^44^. This was relevant due to the importance of increased activity in response to environmental enrichment. For example, pigs negatively affected by environmental husbandry conditions were less active^46^. Negative emotional states in pigs are accompanied by reduced activity and exploration, with greater resting^47^. In addition, previous studies report that an enriched environment (although not with music, but with extra space, peat, and straw in a rack, or herbal compound supplementation) increased exploratory behavior in pigs^48,49^. This behavior is natural and motivated in pigs^50^, and therefore, increased exploration activity is a sign of positive emotions^51^. The state positively occupied was related to exploratory behavior in our study, and an increase in this emotional response was observed during treatment and breaks (the period immediately after stimulation). Therefore, music can generate mood swings that can be sustained after stimulation.

Music is one of the most effective mood induction procedures in experimental psychology^2,46,52^. Additionally, the study of emotions in animals has become a relevant topic, especially for animal welfare. Indeed, animal welfare is defined as "its state as regards its attempts to cope with its environment,"^53^ and as “its emotional evaluation of the outcome.”^54^ In addition, in a review paper on environmental enrichment, the author stated that most studies investigating potential benefits of music on welfare lacked biological relevance, functional meaning, or behavioral control and consequently could not be adequately interpreted^55^. This situation raised doubts about the use of music as a tool for environmental enrichment. However, the present research provided relevant information about the potential use of music and justified the need for further research to verify or contradict previous assumptions.

### Music type effect

Cluster analysis was conducted to identify emotional responses according to the characteristic of the musical stimuli. The number and conformation of each cluster corresponded with harmonic structure considered in the piece’s composition. Therefore, we inferred that these structural elements had an emotional influence on pigs. Although we only considered one of numerous compositional features that can exist in music, in this study, there was a significant relationship with both positive and negative emotions. When using inferential statistics, Cluster 1 (dissonant harmonic structure) was associated with the negative emotional index, whereas Cluster 2 (consonant structure) was associated with the positive emotional index. Cluster 3 (absence of dissonant or consonant structure) did not differ statistically from Cluster 2 in the positive index or from Cluster 1 in the negative index.

Music is comprised of multiple space–time acoustic elements. Neurocognitive processing is required to induce a response in the listener, and the interaction of multiple neuropsychological and emotional functions is required^56,57^. Hence, specific mechanisms that explain how music induces its effects are not completely elucidated. Based on recent findings and theoretical perspectives, various brain areas may be critical for the affective-emotional processing and appreciation of music could be shared in a homologous way with all other mammals^38,40^. Many studies on the processing of musical stimuli have focused mainly on aspects such as rhythm, tone, melody, and harmony^56,58,59^. What was clear from these studies was that many areas of the brain were involved in music processing^60,61^. In this sense, depending on the aspect, quality, or component of the music that is being analyzed (tone, temporal organization, timbre, harmony, melody, etc.), different brain areas will intervene in their interpretation and analysis, making analysis very complex. A variety of research related to both the effects of musical features and the influences of individual differences on human emotional responses has been performed^38,62,63^. Researchers studying emotional impacts of music have not traditionally been concerned with the relationship between the structure of music and its effects on mood in animals. However, musical and acoustic structures of musical forms are importantly related to the perception of emotions^23^; therefore, research on this topic in non-human animals is relevant to understanding emotions related to music.

In this study, we corroborated that music with harmonic structure is related in meaningful ways to pig's emotions. Thus, dissonant music (characteristic of the pieces included in Cluster 1) was related to negative emotions, whereas consonant music (Cluster 2) was associated with positive emotional responses. In the neurocognitive field, potential parallels and differences between harmonic structures have been studied for decades. Theories suggest an implicit effect of hierarchy and structure, tension-relaxation system, and generation of expectations in human listeners^64^. In humans there is a degree of agreement that highly dissonant music tends to be unpleasant and there is a wealth of literature available on musical and psychoacoustic cognition dealing with these issues^65^. In contrast, consonant music was associated with more positive responses; this is not surprising to human listeners, exposed to the Western tonal idiom, a phenomenon that presumably internalized tonal rules of music in their culture^66^. However, in non-human animals, although research in this field is still scarce and mechanisms underlying this type of preference have not yet been clarified, a predilection for consonant music has been suggested in chimpanzees and chickens^32,33^ and current results also indicated it. An adaptive answer to these signals could be suggested as co-evolutionary processes, but this hypothesis would have to be corroborated. Regardless, our results suggest a relevant effect of the harmonic structure of music in non-human animals and, in agreement with what was observed in humans, it may be a prominent element in the design of stimuli and provide information that warrants future research on effects of various types of music, as valuable information that can shed light on biological foundations of music.

The pieces included in Cluster 3 (without harmony), were monodic (1 instrument), and did not generate differentiable emotional responses with other clusters. This presumes remarkable differences between emotional responses and the amount of information that pieces of music contain. The greater quantity of information the pieces had, such as polyphonies with 2 or more instruments (characteristics of the pieces included in Clusters 1 and 2), was related to differentiated emotional responses. A comparative approach with human music perception could provide explanations. For humans, the interest in music is closely related to the speed that one can make sense of what we are hearing. Following music means being able to orient yourself, understand what has been heard, and have a prediction, or an expectation, of where it is going. If our understanding increases proportionally with the speed of musical information, we consider we have enough knowledge to stay current as it unfolds, and we have some confidence that we can anticipate upcoming musical events^67^. High information requires more effort because it has a greater uncertainty^68,69^. In contrast, excessive predictability in musical pieces can induce a lack of interest and attention in the process because they offer no new information^70–72^, which can explain the observations in the emotional responses associated with pieces with a single instrument, corresponding to Cluster 3.

Based on our findings, pigs can respond emotionally to musical stimuli and our data supported the hypothesis that music is a valuable tool for environmental enrichment in animals^73^. However, the influence of constitutive elements of music such as rhythm, melody, or other acoustic parameters on the observed results was not evaluated, and further research is needed to establish the most appropriate musical and acoustic characteristics for acoustic environmental enrichment in pigs. Furthermore, development of effective musical stimuli as an environmental enrichment program requires adaptation of musical pieces to the auditive characteristics and the communication codes of the species of interest, resulting in neurocognitive processing that translates into desired emotional and behavioral responses, based on the premise that enrichment should promote improvements in the quality of life by satisfying behavioral needs.

### Conclusion

Our results demonstrated that nursery pigs exposed to music displayed a wide variety of emotional responses with various affective valences, depending on the harmonic structure of the stimulus. This provided evidence of the potential use of music as an environmental enrichment strategy for this species.

## Methods

### Ethical considerations

All experiments were carried out in compliance with the ARRIVE guidelines (https://arriveguidelines.org), and all methods were performed in accordance with relevant guidelines and regulations. The Ethics Committee in Animal Experimentation of the Universidad de Antioquia (CEEA) authorized all procedures on animals reported herein (Act No. 16, April 10, 2018).

### Study location

The study was conducted at the experimental pig farm of the Universidad of Antioquia (6°26′59.606 N 75°32′37.088 W BH-Mb), Province of Antioquia – Colombia, at an altitude of 2,350 m, with average ambient temperatures of 15 °C, and relative humidity of 70%.

### Litters

Six commercial crossbreed (C29 × PIC 410) litters of 10 to 12 piglets were used as the source of animals, and each litter was considered a replication. Five piglets from each litter were randomly selected for evaluation (n = 30, equal numbers of males and females). At the start of the experiment, piglets were 7 to 9 wk old and 6.5 ± 0.5 kg body weight. They were marked on the back and follow-up evaluations were done without separating them from their group.

### Facilities

Evaluations were performed during the nursery phase. On average, piglets were weaned at 28 d and immediately placed in nursery facilities, housed in 2.5 × 3.0 m pens, with a slightly raised floor of plastic slats and metal bar-walls between pens. Each pen had two nipple drinkers and one hopper feeder. Water and feed were available ad libitum. Lights stayed on from 7:00 to 16:00, and the environmental temperature was ~ 25 °C.

### Musical pieces

A total of 16 (duration, 3 to 5 min each) instrumental original musical pieces were developed for this research. We focused on harmonic structure (a spectral musical feature), as the specific compositional characteristic of each piece. Therefore, the composition of the musical pieces was made determining them as being dissonant, consonant harmonic configuration or absence of harmony.

- Dissonant pieces: we included modal compositions that had a tonal center (i.e., root note) and used Dorian and Phrygian modes. In this system, chords do not have a function; therefore, all chords were equal.
- Consonant pieces: the composition used minor and major tonality, functional harmony, and tonal center (i.e., root note). In tonal harmony, each chord had a function as predominant, dominant or tonic. The function of a predominant chord was to guide towards the dominant chord; therefore, harmony (i.e., the chords) was “functional”. The tonic chord was the “tonal center;” that is, the “center of gravity” around which the other chords gravitated and resolved.
- Pieces without harmony: we included compositions with a single musical instrument.

Therefore, in terms of harmony, pieces were allocated to the following three types:

Group 1. Six dissonant pieces.

Group 2. Six consonant pieces.

Group 3. Four pieces without harmony.

For the composition, musical pieces were recorded in MIDI format in the DAW Ableton live 10 suites, using an Ableton Push 2 controller and a Fishman Triple play MIDI controller device coupled to an electric guitar. The improvisations were then reconfigured and adjusted to musical features, and the scores were written in Sibelius Ultimate® software (AVID 2019). The pieces were exported in MIDI language to the Ableton Live10 suite program. Then, plugins and native virtual instruments and the Kontakt 6 library (Native Instruments) were used. No equalizers, compressors, or spatial effects were considered.

### Experimental design

**Figure 3:**
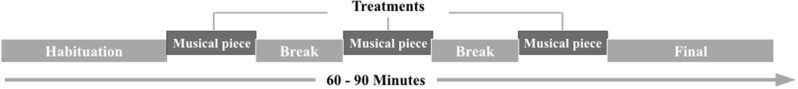
Musical stimulation protocol. Musical pieces used on “treatment” were randomly presented, followed by 3 min “Break” period. Each replicate included a 30 min “habituation” period and another 30 min period after the last piece “Final”.

To avoid habituation to the music, six replications were done, using separate litters for each replication, with a 1-mo interval between them. Pigs spent at least 3 wk in the nursery facilities prior to musical treatments. In each replicate, 30 min before stimulation, a Bose SoundLink Air Digital loudspeaker was installed on-site (this period was considered “habituation”). Then, 4 to 6 musical pieces were randomly presented, including at least one piece of each harmony category, and this was considered the “treatment” period. Between each musical piece, a 3-min interval without music was presented, denominated “break”. The evaluation was extended to 30 min after the exposure to the last musical piece, denominated “Final” period. This arrangement, called a musical stimulation protocol (summarized in Fig. 3), had a maximum duration of 90 min and was started between 9:00 and 10:00 am. Musical pieces for each replicate were randomly selected.

### Evaluation of emotional responses

All repetitions were recorded in videos, the segments of each period (“Habituation,” “Treatments,” “Breaks” and “Final”) were separated and, and the animals' emotional responses were evaluated using the Qualitative Behavioral Assessment (QBA)^74^, a method that has been successfully used for the evaluation of emotions in several species, including horses ^75^; pigs ^76^; buffalos^77^; sheep^78^; dogs^79^; and elephants ^80^. This method is mainly used to evaluate animals’ emotions by integrating their body language information. It captures how individuals interact with their environment by recording “how the animal behaves” instead of “what the animal does.” ^81^ Twenty QBA emotional states were initially included (active, relaxed, fearful, agitated, calm, content, indifferent, frustrated, friendly, bored, playful, positively occupied, lively, inquisitive, irritable, uneasy, sociable, apathetic, happy, distressed). Each state was quantified along a 125 mm visual analog scale that indicated the intensity of each behavioral expression. Then, distances (in mm) from the left margin (minimum) up to the observer's mark for each adjective were measured, thus defining the numeric scores. Video analysis was blind to the observer, with evaluations in randomized order and without sound. To ensure that the observation was blind, for the "habituation" and "final" periods, which had longer recorded time (30 min), a 5-min video fragment (between Minutes 10 and 30 of the period) was taken to be evaluated. Five marked pigs of the litter were selected in each repetition (litter) evaluation, with a total of 30 pigs in the study (n = 30). Each video watching session lasted 3 h. The observer evaluated one video session with an interval of 4 d.

### Intra- and Inter-observer reliability

The general evaluation rating was conducted by only one trained observer, who performed a test–retest reliability evaluation using video clips from a subsample of 20 videos (average of 20 s each). Pearson's correlation coefficient was used to evaluate intra-observer reliability. Intra-observer reliability for each emotional state obtained high values for most of them (r ≥ 0.90; active, fearful, agitated, calm, content, friendly, playful, lively, inquisitive, sociable, happy, uneasy) and moderate (0.50 ≥ r < 0.80) for relaxed, positively occupied, irritable and indifferent. It was also performed an inter-observer reliability with the same subsample, using Pearson’s correlation among two trained observers. Values for all positive emotional states (active, content, friendly, playful, lively, calm, sociable, happy, relaxed, positively occupied) ranged from 0.79 to 0.92, and negative states (inquisitive, fearful, agitated, irritable, uneasy) ranged from 0.85 to 0.92, with all values significant at *P* < 0.001. Thus, the observers reached excellent agreement (r > 0.80) on their scores. The remaining four states (frustrated, apathetic, distressed, bored) were not observed in any evaluation, obtaining a score of "0;" therefore, it was not possible to analyze their correlation coefficients.

### Statistical analyses

Descriptive analyses were obtained for each QBA emotional response using mean and standard deviation. Then, all QBA emotional states were analyzed by applying a principal component analysis (PCA, with correlation matrix and without rotation). This reduced the number of variables by examining the matrix of correlation coefficients between all measurements and infers components, which may help classify the data. To analyze the variation of each PC (emotional index) along the evaluated periods, a general linear mixed model (GLMM) with repeated measures was fitted, including periods (“Habituation”, “Treatments”, “Breaks” and “Final”) as fixed effects.

A cluster analysis was applied to segment musical pieces in groups, according to the emotional responses. A K-means clustering technique was used and supposes that pieces grouped in the same cluster share characteristics between them and differ from pieces grouped in other clusters. To evaluate the variation of emotional indexes (each PC) by the type of music (cluster), a general linear mixed model (GLMM) was fitted, including type of music (cluster) as a fixed effect. The mathematical assumptions about model residuals, specifically normality and homoscedasticity, were tested through visual inference (residual graphs) and hypothesis testing (Shapiro Wilk test and Bartlett or Levene test). A probability level of *P* < 0.05 was chosen as the limit for statistical significance. All analyses were done with R® software (version 4.0.2) through the RStudio integrated development environment. Libraries used for analyses were tidyverse, FactoMineR, factoextra, effectsize, emmeans.

## Data and code availability

The datasets generated during the current study and code implemented for its analysis are available at https://github.com/Julianazapata/Nature-Scientific-Reports.

## Supplementary Information

Supplementary Legends.

Supplementary Video 1.

Supplementary Video 2.

Supplementary Video 3.

Supplementary Video 4.
