# Supplementary material for: Music modulates emotional responses in growing pigs
Source: Sci Rep. 2022 Mar 1;12:3382. doi: 10.1038/s41598-022-07300-6 (PMC8888585; doi:10.1038/s41598-022-07300-6)
Supplement: Supplementary file 1 — Supplementary Legends. [file 41598_2022_7300_MOESM1_ESM.docx]

Videoclip 1. Pigs in a positive emotional state while consonant music is playing.

Videoclip 2. Pigs in a negative emotional state while dissonant music is playing.

Videoclip 3. Pigs during break period. Calm, relaxed and positively occupied states_2.

Videoclip 4. Pigs during final period. Calm and relaxed states.
